# Single Nucleotide Polymorphisms in Starch Biosynthetic Genes Associated With Increased Resistant Starch Concentration in Rice Mutant

**DOI:** 10.3389/fgene.2019.00946

**Published:** 2019-11-15

**Authors:** Selvakumar Gurunathan, Bharathi Raja Ramadoss, Venkataramana Mudili, Chandranayaka Siddaiah, Naveen Kumar Kalagatur, Jutti Rajendran Kannan Bapu, Chakrabhavi Dhananjaya Mohan, Abdulaziz A. Alqarawi, Abeer Hashem, Elsayed Fathi Abd_Allah

**Affiliations:** ^1^Centre for Plant Breeding and Genetics, Tamil Nadu Agricultural University, Coimbatore, India; ^2^DRDO-BU-Centre for Life Sciences, Bharathiar University Campus, Coimbatore, India; ^3^Department of Plant Sciences, University of Saskatchewan, Saskatoon, SK, Canada; ^4^Department of Studies in Biotechnology, University of Mysore, Mysore, India; ^5^Department of Studies in Molecular Biology, University of Mysore, Mysore, India; ^6^Department of Plant Production, College of Food and Agricultural Sciences, King Saud University, Riyadh, Saudi Arabia; ^7^Botany and Microbiology, Department, College of Science, King Saud University, Riyadh, Saudi Arabia

**Keywords:** amylose, resistant starch, starch biosynthesis, rice, mutation/variants

## Abstract

Resistant Starch (RS), plays a crucial role in human health and nutrition by controlling glucose metabolism. RS or dietary fibre content in rice is low because it goes through a variety of process before it is ready for cooking and consumption. Hence, this study was carried out to develop a rice mutant with increased RS. The rice mutant (γ278) with increased RS was developed by utilizing gamma (γ) rays as a mutagen. Mutant γ278 was characterized for mutations in the starch biosynthetic genes *viz*., *GBSSI, SSI, SSIIa, SSIIIa, SBEIa*, and *SBEIIb* to reveal the functional mutations/variations led to high RS content in rice. A total of 31 sequence variants/mutations in six genes were identified. We report the discovery of three deleterious mutation/variants each in *GBSSI, SSIIa*, and *SSIIIa* with the potential to increase RS content in rice. Further, *wild* × *mutant* crosses were made to develop an F_2_ population to study the effect of combination of deleterious mutations. The SNP (*GBSSI*:*ssIIa*:*ssIIIa*) combination responsible for high RS content in F_2_ population was identified and recorded highest amylose content (AC) (26.18%) and RS (8.68%) content. In conclusion, this marker combination will be highly useful to develop a rice variety with increased RS.

## Introduction

Diabetes mellitus (DM) is a major global threat and its prevalence is increasing at an alarming rate worldwide, especially in Asian countries (Cummings and Englyst, 1991; Chan et al., 2009). About 415 million adults suffered with type 2 diabetes mellitus (T2DM) globally during 2015, which is predicted to increase to 642 million by 2040 (Herman, 2017). Apart from obesity and physical inactivity, another major cause of high T2DM prevalence is the consumption of rapidly digestible carbohydrate-rich foods capable of increasing the blood sugar concentration. “Rice is life” for human being especially in the Asian subcontinent, where 90% of world’s rice is grown and consumed by 60% of the population (Khush and Virk, 2000). Although rice is a major cereal globally, efforts to address or enhance the nutritional qualities of rice through crop improvement programs are rare. Rice grain lacks several micronutrients including vitamins and rapidly releases energy after consumption, therefore, has a relatively higher glycemic response compared to other starchy foods (Chassy et al., 2008).

Earlier dietary carbohydrates used to be derived from whole coarse grains loaded with sufficient dietary fibre however, they are currently replaced with refined carbohydrates devoid of any dietary fibre, e.g. modern day rice resulting from advanced milling technology (Chattopadhyay, 2005). It is known that refined carbohydrate-rich diet enhances the plasma glucose level, insulin, triglycerides (TAG), and non-esterified fatty acid (NEFA) thus play a significant role to insulin resistance (Wolever and Mehling, 2003). Due to lifestyle changes, cells of the body did not respond properly to the hormone insulin which precedes the development/onset of T2DM. In a recent systemic review based on four prospective cohort studies with a total of 13,284 incident cases of T2DM among 352,384 Asian participants, Hu et al. (2012) concluded that the higher consumption of white rice was associated with a significant increase T2DM risk. In contradiction, the scientific investigation from Miller et al. (1992) indicated that all processed forms of rice (white, brown, or parboiled) fell under the category of high-glycemic index (GI foods). This signifies the necessity to develop rice variety(ies) with reduced starch hydrolysis or glycemic index utilizing molecular breeding approaches.

Starch is a complex glucose polymer stored as distinct water insoluble granules in rice grains (∼90%) primarily composed of one-quarter amylose and three-quarters amylopectin (Ramadoss et al., 2019). Starch is divided into three fractions based on the enzymatic hydrolysis, readily digestible (RDS), slowly digestible (SDS), and resistant starch (RS) (Englyst and Hudson, 1996). Amylose, an important determinant of RS formation as it showed a positive correlation with amylose (Ramadoss et al., 2015), is more resistant to enzymatic hydrolysis compared to its counterpart amylopectin (Leeman et al., 2006; Lehmann and Robin, 2007) and therefore, is being widely used to enhance the RS content of processed foods. Therefore, amylose concentration in starch can be targeted to improve RS content in the rice grains. Increasing amylose and RS content in rice endosperm is envisaged as a potential target to enhance its starch quality and promote human health and nutrition (Fuentes-Zaragoza et al., 2010). RS has a significantly lower hydrolysis index compared to other fractions of starch and free glucose moieties thus assist to normalize blood sugar levels in diabetic people. In addition, RS is fermented by large intestinal microflora into short chain fatty acids promoting the growth of beneficial bacteria and lowering the pH of the intestinal microenvironment thus alleviating the risk of gastrointestinal (GI) disorders. Extensive studies also have shown that RS possess physiological functions like those of dietary fiber (Eerlingen and Delcour, 1995).

Three genetic mechanisms have been described to increase amylose and RS content in cereals. Interestingly, the specific nature and impact of each mechanism can differ among cereals despite their close relatedness. The first mechanism includes overexpression of granule-bound starch synthase I (*GBSSI*) to increase amylose content (AC) (Flipse et al., 1996; Umemoto et al., 2004). The second mechanism to increase amylose accumulation in the developing grain is to down-regulate *SSIIa* or *SSIIIa* activity and decease amylopectin biosynthesis. Moreover, suppressed expression of *SSIIIa* has been associated with up-regulation of *GBSSI* and therefore, to increased amylose content (Umemoto et al., 2008; Sestili et al., 2012). The most prominent third mechanism, to increase apparent AC in cereals, targets suppression of major starch branching enzyme (*SBEIIb)* activity as structural changes in starch granule may have a great impact on RS content, particle size distribution and crystallinity. Mutation in starch synthases (*SSIa*, *SSIIa*, and *SSIIIa)* produce altered amylopectin chain length distribution in rice endosperm (Fujita et al., 2007). The suppression of starch branching enzyme *SBEI* alone has either no detectable effect (Regina et al., 2004), or only a minor effect on amylopectin chain length. Moreover, suppression of *SBEIIb* leads to elevated AC in maize and rice with altered amylopectin chain length distribution (Butardo et al., 2011) and suppression of both *SBEIIb* and *SBEI* in rice led to high AC (Zhu et al., 2012). Previous studies have been focused on the identification of mutations and characterization to understand the role of individual mutations in starch biosynthesis. This study was aimed to discover mutations responsible for increased RS content in the identified mutant line γ278 and to understand the role/contribution of individual mutations and/or combination of mutation towards trait improvement. Consequently, the present study characterized a high RS rice mutant line (mutant γ278), developed through gamma mutagenesis and biochemical analysis. The developed mutant was characterized for key candidate genes in the starch biosynthesis (*GBSSI, SSI, SSIIa, SSIIIa, SBEIa,* and *SBEIIb*) which is responsible for high RS content and to find out the sequence variants/mutations between the high RS mutant (γ278) and its wild type ADT43. Further, we analyzed the functional sequence variants/mutations for their contribution on the trait of interest through the development of segregating population (F_2_) of ”*wild type* × *mutant*” cross and revealed the role of gene-specific mutations towards an increase in rice RS content.

## Materials and Methods

### Plant Material and Mutational Dose Determination and Screening

The cultivar ADT 43 was chosen for inducing mutations which is a short duration variety derived from a cross between IR 50 and Improved White Ponni (IWP). To create an ADT43 rice mutant population, gamma radiation (Tamil Nadu Agricultural University-Radiation facility) was used and LD_50_ value (250 Gy) was determined using kill curve analysis. Fixed LD_50_ was used to treat and to develop the 5,000 M_1_ plants (Agasimani et al., 2013). Then the 3,000 M_2_ families were raised and three plants from each family have been tagged. The seeds were harvested in a single plant basis and a portion of seeds was processed to white rice and used for biochemical analysis (data not shown). The RS content of M_2_ to M_6_ generation was estimated on dry weight basis following Goni et al. (1996) using the Megazyme RS assay kit (Cat#K-RSTAR; Megazyme International Ireland Ltd., Ireland). Identified mutant (γ278) line with increased RS was used for further molecular studies to identify the functional mutations.

### DNA Isolation

Total genomic DNA was extracted from the leaves using DNeasy 96 Plant kit (Qiagen, Valencia, CA, USA) according to the manufacturer’s instructions.

### Candidate Gene Selection and Gene Model Construction

The existing literature was reviewed to select the candidate genes associated with starch composition and quality (Rahman et al., 2000; Nakamura, 2002; Hirose et al., 2006; Waters and Henry, 2007). Based on these literatures, a total of six key candidate starch synthesis pathway genes including *Granule Bound Starch Synthase I* (*GBSSI*), *Starch Synthase I (SSI), Starch Synthase IIa (SSIIa), Starch Synthase IIIa (SSIIIa), Starch Branching Enzyme Ia (SBEIa),* and *Starch Branching Enzyme IIb (SBEIIb)* were undertaken to reveal the influence of target gene mutations in RS enhancement (**Table 1**). Targeted gene sequences were retrieved from the NCBI genome database (http://www.ncbi.nlm.nih.gov/Genbank) gene model was constructed by using Splign (https://www.ncbi.nlm.nih.gov/sutils/splign/splign.cgi) bioinformatics pipeline.

**Table 1:** Details of selected candidate genes with their function used in this study.

S no.	Gene	ID of mRNA	Genomic ID	Size of the gene (kb)	Function
1. 2. 3. 4. 5. 6.	Granule Bound Starch Synthase (*GBSSI*) Starch Synthase I (*SSI*) Starch Synthase IIa (*SSIIa*) Starch Synthase IIIa (*SSIIIa*) Starch Branching Enzyme Ia (*SBEIa*) Starch Branching Enzyme IIb (*SBEIIb*)	AB425323.1 AK109458.1 AB115918.1 GQ151020.1 AK065121.1 D16201.1	NC_008399.2 NC_008399.2 NC_008399.2 AK061604 NC_008399.2 NC_008395.2	3.479 6.815 4.419 11.083 4.745 10.899	Amylose biosynthesis Amylopectin synthesis Amylopectin synthesis Amylopectin synthesis Amylopectin synthesis Amylopectin synthesis

### Target Gene Amplification and Mutation Identification

By using the target gene sequences and expressed sequence tags (EST), a total of seven primer pairs were designed using Primer3 software (Primer3; https://www.ncbi.nlm.nih.gov/tools/primer-blast/), and used to amplify the target genes. Primer sequences and target amplicon lengths were presented in **Table 2**. PCR reactions (50 μl) were performed using 10 μl of 5× long AMP Taq Reaction buffer, 1.5 μl of dNTPs (10 mM), 2 μl of each forward and reverse primer (10 μM), 2μl of DMSO, 5 μl of DNA (50ng/μl), 2 μl of Long Amp^®^ Taq polymerase (5 unit), and 25.5 μl sterile water. Step down PCR cycling was performed using ABI 2720 (Applied Biosystems, Foster City, CA) thermal cycler.

**Table 2:** Details of gene specific primers designed to amplify selected candidate genes.

S no.	Gene	Order	Primer Sequence (5′ to 3′)	Amplicon size (Kb)
1	Granule Bound Starch Synthase (*GBSSI*)	F	TTCATCTGATCTGCTCAAAG	5.525
		R	CCAGAAGAGTACAACATCAAAC	
2	Starch Synthase I (*SSI*)	F	ATCACTTCACAAACCCATAAC	7.604
		R	GAAAGACAGGAAGATTGAGG	
3	Starch Synthase IIa (*SSIIa*)	F	AAAGTAACTCGCTTCTGGAG	5.112
		R	AAGAAGTAACATCGCATCAAT	
4	Starch Synthase IIIa (*SSIIIa*)	F	CTTCTATGCCCTCGGAGCAG	11.018
		R	ACATAGCTGATACATACTCCCA	
5	Starch Branching Enzyme Ia (*SBEIa*)	F	GGTGACTGTTGTGGAGGAGG	4.730
		R	CGTCAGAAGACCGAAACACA	
6	Starch Branching Enzyme IIb (*SBEIIb*)	F1	AGCACAGGAGTAGCAAGTAG	6.770
		R1	AACTACTGCATCAGCATCAG	
		F2	GCACCAAGTAGTCGTTTCGG	6.508
		R2	TGGAGCATAGACAACGCAGG	

F, Forward primer; R, Reverse primers.

### Site-Specific Gene Sequencing and Mutant Validation

Upon successive amplification of target genes, site-specific target gene sequencing was carried out using Ion Torrent Next Generation Sequencing (NGS) platform with 1Gb data output using Ion 316™ Chip by following the manufacturer’s instruction. After the sequencing of libraries, aligning of sequencing information were carried out by using Torrent Suite 1.5 with wild type (ADT 43) reference sequences. After sequence alignment, variant caller bioinformatic pipeline was used with selected parameters such as min–max distance, mismatch cost, length fraction, and similarity were selected to pull out rare SNPs from the aligned sequence contigs in comparison with their wild type reference sequences. The minimum variant frequency of 0.5 and minimum coverage of 20 was used to detect rare SNPs which gives variants on or above 0.5% were considered as SNPs.

Identified SNP sequence variants were analyzed by the PARSESNP program (Taylor and Greene, 2003), which provides information on the location along with the details about amino acid changes and severity of mutations. SIFT (http://sift.jcvi.org/www/SIFT_seq_submit2.html.) a web-based tool which predicts whether amino acid substitution affects protein function and structure based on sequence homology and the physical properties of amino acids (Ng and Henikoff, 2003) was employed to discover functional mutations. The predicted SIFT score ranges from 0 to 1. The amino acid substitution is predicted to be damaging if the score is <0.05 and tolerated if the score is >0.05.

### Starch Structural Analysis

Starch structural analysis was carried out using a scanning electron microscope (SEM) (FEI, USA) as described by Fujita et al. (2003).

### Trait Association and SNP Analysis Through Molecular Breeding

#### Wild Type × Mutant Crosses

For trait association, F_1_ plants were developed by crossing γ278 with wild type ADT 43. Five F_1_ plants were selected and tagged for SNP genotyping with G→A transition in *SSIIa* gene to identify the true hybrid. SNP genotyping was carried out using KASPar technology (LGC genomics, Ipswich, UK). The true F_1_ plants (possessing the SNP markers in a heterozygous state) were self-pollinated and produced 538 F_2_ plants.

#### SNP Genotyping of F_2_ of “*Wild* × *Mutant*” Cross

Mutant segregation analysis and their contribution towards the trait of interest were analyzed with 192 F_2_ plants of the “*wild type* × *mutant*” crosses. The observed and expected distribution of mutation in the F_2_ plants were analyzed using simple excel workbook suggested by Montoliu (2012). SNP genotyping of F_2_ plants was carried out using KASPar technology (LGC genomics, Ipswich, UK) to determine the SNPs. Seeds of each tagged plants were harvested and stored for further study.

#### Estimation of AC and RS Concentration

The RS **concentration** of grain samples from the 28 F_2_ plants representing various allelic combination was estimated along with their parent traits including ADT43 and γ278 (Goni et al., 1996) using the Megazyme RS analysis kit. AC estimation was carried out using the method explained earlier (Raja et al., 2017b).

## Results

### Mutational Dose Determination and Screening

LD_50_ value of γ-radiation was determined as 250 Gray for ADT 43 rice variety. Fixed LD_50_ was used to treat and to develop the mutant population (Agasimani et al., 2013). The M_2_ mutant population was analyzed for their RS and AC (data not shown). Results of RS estimation further revealed that there is a significant change in the RS concentration ranged from 3.61 ± 0.4% to 6.62 ± 0.4% (data not shown). Further F_2_ analysis revealed that the rice mutant named γ278 showed a maximum RS concentration of 8.63 ± 0.53%. Additional molecular characterization studies were undertaken to reveal the genes and its mutations responsible for quantitative up-gradation of RS concentration in the identified rice mutant line γ278.

### Target Gene Amplification and Mutation Identification

**Figure 1:**
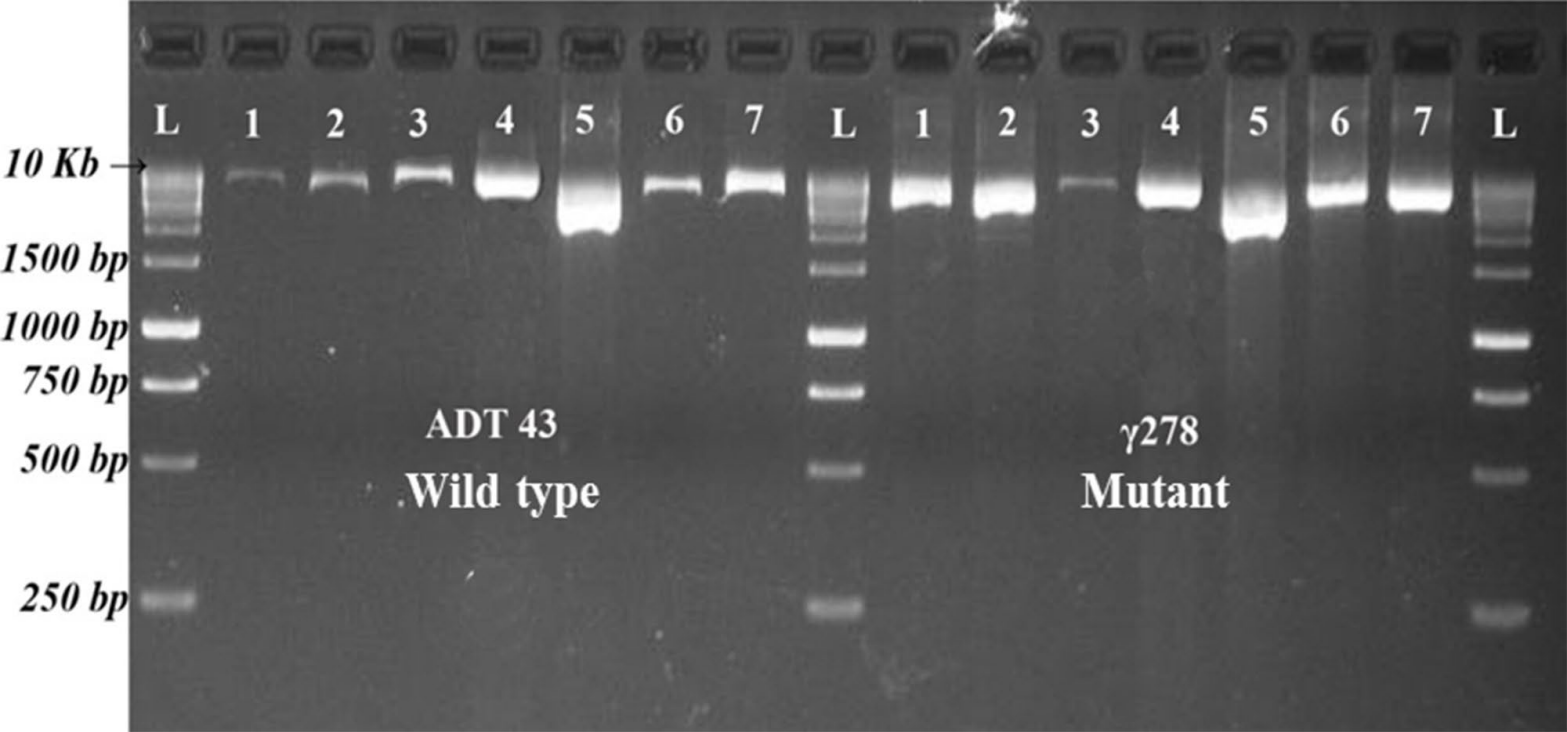
Resolved PCR products of candidate genes in ADT43 and γ278. L = Size Marker, 1 = *GBSS I*, 2 = *SS IIa*, 3 = *SS IIIa*, 4 = *SS I*, 5 = *SBE Ia*, 6 = *SBE IIb* Fragment 1, and 7 = *SBE IIb* Fragment 2.

PCR conditions were optimized for selective amplification of target genes. Various PCR cycling conditions and master mix composition were standardized for the amplification of different candidate gene in the mutant and wild type genotype. After successful amplification (**Figure 1**), PCR products of wild type and mutants were pooled across the target genes and then barcoded for easy interpretation of the sequence analysis. The average read depths were recorded in the range of 432 to 969. Sequencing results revealed that target gene *SBEIa* recorded with a maximum average read depth of 969 followed by *SSIIa* (879), *GBSSI* (824), *SSIIIa* (709), *SSI* (689), and *SBEIIb* (432). Mutation with higher confidence levels in the target genes were identified using variant caller and the mutations were filtered with the quality score of 60 and above. From 47.28 kb of six candidate genes, 31 (28 SNPs and three Indels) variants were identified (**Table S1**). Eight variants were discovered in *SSI* (seven SNPs and one Indel), *SSIIIa* (eight SNPs) followed by five each in *GBBSI* (four SNPs and one Indel), and *SBEIIb* (four SNPs and one Indel). Three SNP variants were discovered in *SSIIa*. *SBEIa* recorded two SNP variants and all of them were found to be in intron.

Further position analysis revealed that five sequence variants were identified in *GBSSI*, two were in exon region and rest was detected in the intron region. In the case of *SSIIa*, out of three sequence variants identified, two were found in exon regions. Interestingly, a total of eight sequence variants were found in *SSIIIa*, out of which four were found in exon regions and the rest of them were in the intron regions.

### Functional Validation of SNPs

Functional analysis of the exon residing mutations indicated that 62.5% were silent and 32.5% were deleterious mutations. The discovered exon residing mutations in the γ278 along with their SIFT score were furnished in **Table 3**. Two exon residing mutations observed in the GBSS I gene, SIFT analysis of exon residing SNPs revealed that only one SNP at position 2078 (C→T) of the reference sequence in ninth exon of the gene is a functional mutation leading to Proline to Serine substitution at 415th amino acid residue with a SIFT score of 0.00. This functional mutation in C→T (2078) lead to the creation of restriction site *Hpy188I* and deletion of *ApaI, AsuI, DraII, HaeIII*, and *NlaIV* restriction sites further indicated the possibility of generating CAPS/dCAPS marker. On the other hand, the sequence variation T→C (1804) was found to be a silent variation with the SIFT score of 1.00, as it did not cause any amino acid change.

**Table 3:** Deleterious mutations identified in the selected candidate genes.

S. no.	Candidate gene	Nucleotide change^a^	Effect on protein sequence^b^	Restriction site		SIFT Score	Effect of mutation
				Gained in variant	Lost from reference		
1	*GBSSI*	T1804C	P362=	*BseYI*	*MaeI*	1.00	Silent
		C2078T*	P415S	*Hpy188I*	*ApaI, AsuI, DraII, HaeIII, NlaIV*	0.00	Deleterious
2	*SSIIa*	G3797A*	G604S	*Eco57MI, GsuI*	*HpaII*	0.00	Deleterious
		G3901T	G638=	*–*	*–*	–	Silent
3	*SSIIIa*	C1615T*	A195V	*–*	*AciI, SfaNI*	0.00	Deleterious
		T2276C	H415=	*–*	*AvaIII, NlaIII*	1.00	Silent
		C3135A	R702=	*–*	*–*	1.00	Silent
		G5515A	L1256=	*–*	*–*	1.00	Silent

a The letter on the left side of the numeral specifies the wild type nucleotide; letter on the right side of the numeral specifies the altered nucleotide; numeral specifies the base pair position of the nucleotide change with respect to the gene sequence.

b Signifies a synonymous change; the numeral specifies the residual number of the amino acid based on the gene model; letter on the left of the numeral specifies wild type amino acid; letter on the right side of the numeral specifies the altered amino acid.

* Indicates that the nucleotide change resulted in deleterious mutation.

The two identified exon variants (G→A at 3797 and G→T at 3901) of *SSIIa*, were found in the region of eighth exon. The mutation G→A triggered an amino acid substitution Glycine to Serine at the position of 604 in protein sequence and caused the intolerant mutation and showed the deleterious effect on the protein function with a sift score of 0.00. On the other hand, the mutational sequence variation G→T at 3901 did not yield any amino acid change in the target protein sequence.

Four exon residing mutations (position of nucleotide change) C→T (1615), T→C (2276), C→A (3135), and G→A (5515) were identified in *SSIIIa*. Among them, three found in the region of third exon and rest was present in the fifth exon. Out of four mutations discovered in the third exon, C→T (1615) change resulted in amino acid substitution of Alanine to Valine at the position of 195 and showed deleterious effect with a SIFT score of 0.00 and rest of the variants did not show any significant change in the amino acid sequence. C→T variation further leads to the deletion of *AciI* and *SfaNI* restriction sites without the addition of new restriction site.

### Phenotypic Discrimination and Grain Structural Analysis

**Figure 2:**
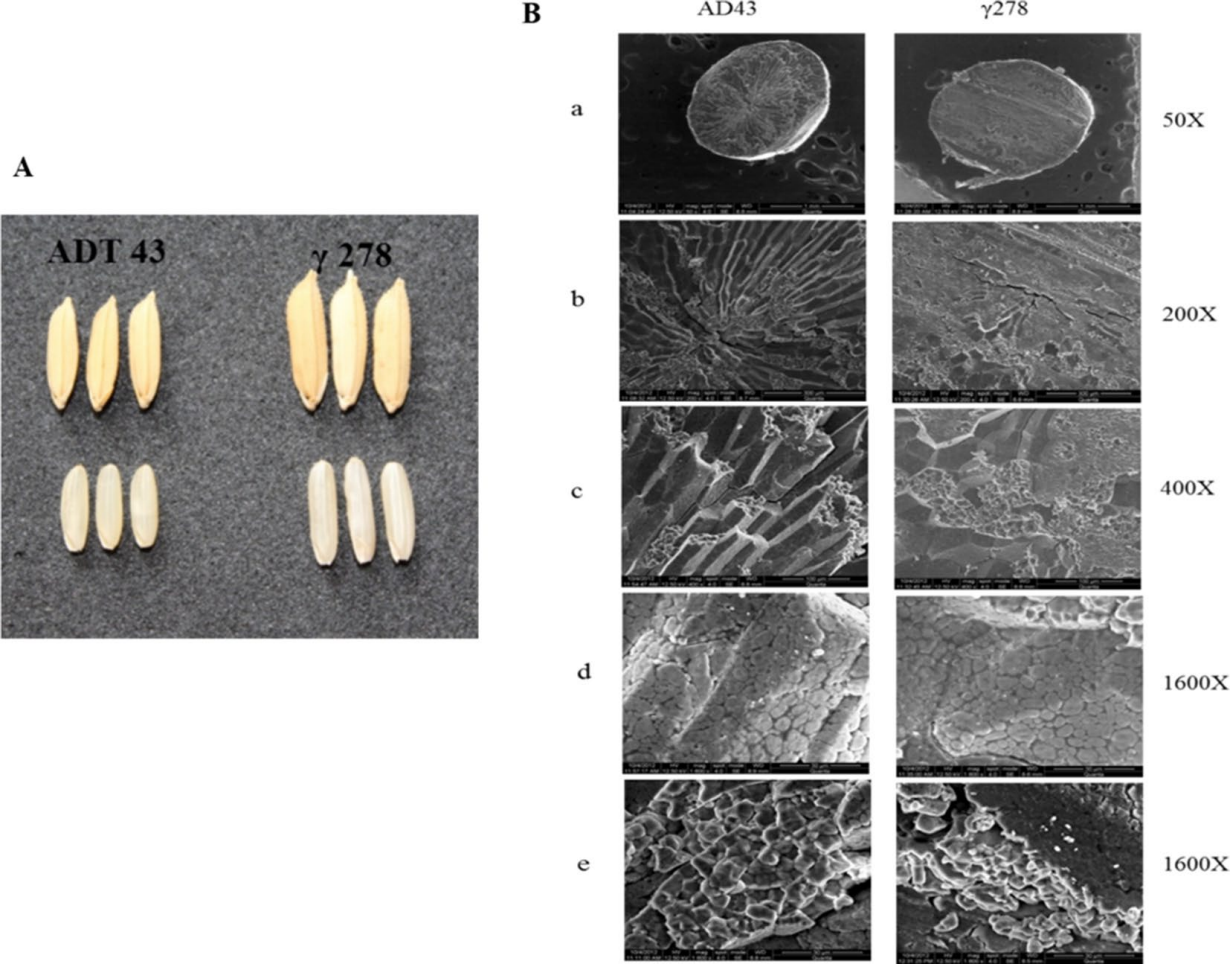
Phenotypic and starch grain variation in the wild type and mutant grains. **(A)** Phenotypic variation of mutant and wild type genotype. **(B)** Scanning electron micrograph of starch in the mutant and wild type grains in different magnification (a-e).

To study the effect of mutations in the grain phenotype, observations were recorded on morphology based on visual observation a significant difference in the mutant and wild type grains (**Figure 2A**). Also, to study the effect of mutation on starch structural properties, SEM analysis of mutant and wild type grains were studied (**Figure 2B**). SEM analysis revealed that there is a significant structural alteration in the starch granule arrangement of mutant grain in comparison with its wild type (**Figure 2B a, b, c**). The structural changes were recorded as granules with loosely packed in nature and presence of numerous airspaces between the granules (**Figure 2B d**) in mutant grain (γ278), whereas, fewer airspaces with compact granule arrangement were observed in the wild type (ADT43). Furthermore, γ278 was recorded with smaller, round-shaped starch granules accumulated in the amyloplast of endosperm cells, in contrast to the sharp-edged polygonal granules in the ADT-43 (**Figure 2B e**). Moreover, the phenotypic observations revealed that a higher proportion of smaller granule size coupled with white core belly which is absent in the wild type genotype ADT 43.

### Role of Mutation in RS Enhancement

The role of identified mutations in RS enhancement as individually and as in combination was determined by employing “*wild type* × *mutant*” hybridization to generate the F_1_ population. The true F_1_ plants were confirmed through KASPar–SNP genotyping. A total of 192 F_2_ plants were taken for genotyping to study the effect of individual mutation. Interestingly, all the studied mutations are in line with the Mendelian segregation ratio (1:2:1) in 192 F_2_ plants (**Table 4**). Amylose and RS concentration analysis of F_2_ plants harboring the 8 pre-determined mutant SNP marker combinations revealed that out of eight homozygous marker combinations (**Table 5**), two of them recorded with higher mean RS content than the mutant γ278, whereas five of them recorded intermediate mean RS concentration between wild type and mutant γ278. Moreover, the highest mean concentration of amylose (26.18%) and RS (8.68%) were recorded in F2 plants with the marker combinations of CC : AA:TT among the target genes of *GBSSI: SSIIa: SSIIIa* respectively and followed by CC : GG:TT combination which is recorded 25.23% of AC and 7.79% of RS concentration (**Table 5**). The marker combination TT : AA:TT recorded with RS concentration of 7.03% even it having lower AC than the marker combinations CC : AA:CC (RS — 6.19%) and TT : GG:TT (RS — 5.74%). The lowest value for AC (20.89%) and RS concentration (2.27%) were recorded in the marker combinations of TT : GG:CC.

**Table 4:** Chi Square table representation of SNP segregation in target genes.

*GBSSI*					*SSIIa*				*SSIIIa*			
	Expected	Observed	O–E	Chi square value	Expected	Observed	O–E	Chi square value	Expected	Observed	O–E	Chi square value
Wild	48 (1)	58	10	2.08	48 (1)	51	3	0.18	48 (1)	49	1	0.02
Heterozygous	96 (2)	99	3	0.09	96 (2)	102	6	0.37	96 (2)	99	3	0.09
Mutant	48 (1)	35	−13	3.52	48 (1)	39	−9	1.68	48 (1)	44	−4	0.33
Total	192	192	0	5.69^NS^	192	192	0	2.25^NS^	192	192	0	0.44 ^NS^

^NS^ No significant deviation.

**Table 5:** Amylose and RS concentrations of selected F2 plants with different combinations of mutations discovered in this study.

S no.	Marker combinations	Number of plants	Well position	Plant	AC (%)	Mean AC (%)	RS (%)	Mean RS (%)
1.	CC : GG:CC *GBSSI: SSIIa: SSIIIa*	4	H01	F2-8	22.36 ± 0.6	22.35 ± 0.5	3.90 ± 0.2	3.80 ± 0.2
			F06	F2-46	22.79 ± 0.8		3.81 ± 0.1	
			B02	F2-106	22.11 ± 0.5		3.68 ± 0.3	
			H05	F2-136	22.14 ± 0.4		3.84 ± 0.2	
2.	CC : GG:TT *GBSSI: SSIIa: ssIIIa*	5	H06	F2-48	25.25 ± 0.3	25.23 ± 0.4	7.82 ± 0.4	7.79 ± 0.3
			G08	F2-63	25.16 ± 0.7		7.79 ± 0.3	
			F09	F2-70	25.31 ± 0.4		7.64 ± 0.5	
			B12	F2-90	25.21 ± 0.2		7.94 ± 0.2	
			H03	F2-120	25.23 ± 0.4		7.77 ± 0.1	
3.	*CC : AA:TT* *GBSSI: ssIIa: ssIIIa*	2	E05	F2-133	26.03 ± 0.5	26.18 ± 0.6	8.71 ± 0.4	8.68 ± 0.3
			D09	F2-164	26.34 ± 0.8		8.65 ± 0.3	
4.	CC : AA:CC *GBSSI: ssIIa: SSIIIa*	6	F08	F2-62	24.89 ± 0.2	24.61 ± 0.4	6.23 ± 0.2	6.19 ± 0.4
			D09	F2-68	24.46 ± 0.4		6.31 ± 0.3	
			D03	F2-116	24.42 ± 0.6		6.04 ± 0.4	
			G07	F2-151	24.67 ± 0.4		6.27 ± 0.2	
			C10	F2-171	24.48 ± 0.6		6.13 ± 0.5	
			C12	F2-187	24.74 ± 0.5		6.18 ± 0.6	
5.	TT : AA:CC *gbssI: ssIIa: SSIIIa*	2	F10	F2-174	23.34 ± 0.3	23.42 ± 0.4	5.75 ± 0.4	5.58 ± 0.4
			E11	F2-181	23.51 ± 0.5		5.42 ± 0.5	
6.	TT : GG:CC *gbssI: SSIIa: SSIIIa*	4	A02	F2-9	20.68 ± 0.2	20.89 ± 0.3	2.44 ± 0.1	2.27 ± 0.3
			C08	F2-59	21.10 ± 0.2		2.01 ± 0.5	
			D11	F2-84	20.87 ± 0.5		2.24 ± 0.3	
			H11	F2-184	20.93 ± 0.3		2.41 ± 0.4	
7.	TT : GG:TT *gbssI: SSIIa: ssIIIa*	2	G08	F2-159	24.33 ± 0.5	24.40 ± 0.4	5.67 ± 0.5	5.74 ± 0.4
			H12	F2-192	24.47 ± 0.3		5.81 ± 0.4	
8.	TT : AA:TT *gbssI: ssIIa: ssIIIa*	3	F01	F2-6	23.51 ± 0.6	23.48 ± 0.4	6.98 ± .05	7.03 ± 0.5
			D01	F2-100	23.43 ± 0.4		7.10 ± 0.4	
			G01	F2-103	23.52 ± 0.2		7.02 ± 0.5	
9.	ADT 43					22.17 ± 0.2		3.61 ± 0.4
10.	γ 278					23.40 ± 0.3		7.26 ± 0.4

## Discussion

The existing cereals contributing a major portion to the daily diet lack the appropriate concentration of RS to meet out the per day requirement, therefore, daily intake of RS is much lesser than the minimum recommended levels (6 g *per* meal) for health benefits (Dian et al., 2005). Currently, researchers and nutritionists have shown increased emphasis on research exploring the starches resistant to human digestive enzymes. Rice is one of the major staple cereals with low RS concentration utilized among the globe. Introducing an important trait such as RS in rice grains will assist to prepare food products rich in RS/dietary fibre, alleviating the burden of T2DM, and improve the socio-economic condition of the society. Earlier researchers postulated that RS concentration is positively correlated with amylose concentration (AC) in cereal crops (Benmoussa et al., 2007; Sang et al., 2008; Ramadoss et al., 2015) however, it would be disadvantageous to the cooking quality and palatability. Alternatively, other starch properties such as granule size, architecture, crystalline pattern, the degree of crystallinity, surface pores, or channels and degree of polymerization also influence starch digestibility (Tester et al., 2006; Noda et al., 2008). However, amylose concentration and amylopectin chain length variation influence the formation of RS concentration in rice endosperm (Fujita et al., 2007; Nakamura et al., 2010). Mutation/nucleotide changes in starch biosynthetic genes (*GBSSI, SSI, SSIIa, SSIIIa, SBEIa*, and *SBEIIb*) lead to variation in the starch quality/quantity in rice endosperm. Therefore, the present study was undertaken to study the effect of allelic variation in the target genes which contribute towards the enhancement of RS concentration in rice endosperm.

Exposing seeds to the radiation is one of the well-known approaches in mutation breeding. To know the effect of radiation on the change of gene structure and enzyme function, we characterized important candidate genes which are responsible for increasing the RS fraction of starch in the rice grains by adopting PCR and re-sequencing mutation/variant discovery method through *in silico* and *in vivo* approaches.

NGS sequencing results together with PARSESNP analysis revealed that the mutation C→T (2078) might cause an amino acid substitution of proline to serine at the 415 position in amino acid sequence of *GBSSI* gene. Mutations in *GBSSI* showed reduced activity by affecting starch-binding capacity (Liu et al., 2014), and its ADP–glucose-binding capability or its protein stability. Moreover, Delrue et al. (1992) reported that the Chlamydomonas mutant lacking *GBSSI* activity generated amylose free starch with the altered structure of amylopectin. Therefore, a deleterious mutation in *GBSSI* in the present study was expected to have lesser AC in γ278 mutant than wild type, but surprisingly the determined AC in γ278 was found to be more than the corresponding wild type. This could be explained by the mutations in the key candidate genes (*SSIIa* and *SSIIIa*) of amylopectin biosynthesis that might be responsible for the altered ratio of amylose and amylopectin which may lead to an incremental effect on AC than amylopectin in the total starch.

Mutation G→A (3797) in *SSIIa* caused an amino acid substitution of Glycine to Serine at the position of 604 in the mutant γ278. The same amino acid substitution (G604S) was reported to result in amylopectin chain length variation between *japonica* (Serine) and *indica* (Glycine) types (Nakamura et al., 2005). Among the four exon residing mutations identified in *SSIIIa* gene, the variant C→T (1615) cause amino acid substitution of Alanine to Valine at the position of 195 with deleterious in nature. Raja et al. (2017a) also reported the same mutation in rice found to have increased RS concentration. Previous reports also indicated that the loss of *SSIIIa* function leads to accumulation of white-core ﬂoury endosperm in rice (Fujita et al., 2007). Further, it was found that the accumulation of white core floury endosperm is due to enrichment of amylose and the loose packing of starch granules in the mutants (Fujita et al., 2007). Similar results were also reported by Yoshii (2000) and Nagato and Ebata (1958).

Upon successive mutant detection, SNP genotyping and trait association analysis was carried out to reveal the role of each mutation with respective to RS enhancement in the wild *type* × *mutant* derived F_2_ population. Highest RS concentration of 8.68% was recorded in the marker combination of *GBSSI*: *ssIIa*: *ssIIIa.* Here we discovered wild type *GBSSI* and defective *ssIIa* and *ssIIIa* enhanced the RS level up to 8.68%. Zhou et al. (2016) reported active *GBSSI* and defective *SSIIIa* leads to high RS formation in rice endosperm. Marker combination *GBSSI : SSIIa*:*ssIIIa* was recorded RS value of 7.79%. Mutation free GBSS*I* resulted in enhanced AC, conversely mutations in *SSIIa* and *SSIIIa* leads to the formation of more short chains in amylopectin led to enhanced RS concentration. Similar results were also reported in rice by Sasaki et al. (2009). On the other hand, in spite of its lower AC, the marker combination of *gbssI:ssIIa:ssIIIa* recorded with 7.03% RS, than the marker combinations of *GBSSI:ssIIa : SSIIIa* (RS — 6.19%) and *gbssI: SSIIa: ssIIIa* (RS — 5.74%) with high AC. The marker combination *gbssI:ssIIa:ssIIIa* having mutant SNP in all the three genes lead to decreased AC and in-turn increasing the significant structural modifications by increasing the α -1-6 linkages which are less prone to α-amylose (Regina et al., 2015). Similar kind of findings were reported by Zihua et al. (2007) who reported that the concentrated α-1,6 linkages in amylopectin lead to the decrease in overall enzyme digestion rate. Because of mutant *gbssI*, lowest recorded AC (20.89%) and RS concentration (2.27%) were analyzed in marker combinations of *gbssI:SSIIa : SSIIIa*.

## Conclusion

Among the analyzed marker combinations in the present study, wild type *GBSSI* with mutant gene alleles of *ssIIa* and *ssIIIa* will be the promising combinations to screen a large number of mutant/germplasm lines for enhanced RS in rice. This marker combination can also be used to develop a cultivar with increased RS concentration through marker-assisted breeding. High RS line developed in this study can be used to manage the global burden of T2DM lines. The outcomes of the present study may lead to propose a novel insight into the development of high yield rice varieties and hybrids with value-added properties in the near future.

## Data Availability Statement

All datasets generated for this study are included in the article/**Supplementary Material**.

## Author Contributions

SG performed all the experiments and data analysis. BR helped in execution of all the experiments. VM, CS, and NK helped in execution of bioinformatic and structural analysis. JB and SG together conceptualized this study. SG, BR, CM, AA, AH, and EA drafted the manuscript with the input from JB. All authors read and approved the final manuscript.

## Funding

Financial support for this study was provided by the pioneer international hi-bred student fellowship of the pioneer international USA to the first author to carry out this work as a part of doctoral program.

## Conflict of Interest

The authors declare that the research was conducted in the absence of any commercial or financial relationships that could be construed as a potential conflict of interest.
