# Supplementary material for: Single Nucleotide Polymorphisms in Starch Biosynthetic Genes Associated With Increased Resistant Starch Concentration in Rice Mutant
Source: Front Genet. 2019 Nov 15;10:946. doi: 10.3389/fgene.2019.00946 (PMC6872638; doi:10.3389/fgene.2019.00946)
Supplement: Supplementary file 1 [file Table_1.docx]

**Table S1:** List of mutations discovered in the selected candidate genes

| **S.No** | **Gene name** | **Nucleotide change position** | **Reference base** | **Called base** | **Type of mutation** | **Position of mutation** | **Read depth** | **Frequencies** | **Q call** |
| --- | --- | --- | --- | --- | --- | --- | --- | --- | --- |
| 1. | *GBSS1* | 1019 | C | - | Indel | Intron | 1000 | 99.8/0.2 | 60 |
| 2. | *GBSS1* | 1097 | C | T | SNP | Intron | 1000 | 93.8/6.2 | 60 |
| 3. | *GBSS1* | 1804 | T | C | SNP | Exon | 985 | 98.5/1.5 | 60 |
| 4. | *GBSSI* | 2078 | C | T | SNP | Exon | 977 | 84.4/15.6 | 60 |
| 5. | *GBSSI* | 2221 | G | A | SNP | Intron | 953 | 94.0/6.0 | 60 |
| 6. | *SSI* | 1664 | T | C | SNP | Intron | 577 | 95.6/4.4 | 60 |
| 7. | *SSI* | 2947 | G | A | SNP | Intron | 530 | 98.0/2.0 | 60 |
| 8. | *SSI* | 2996 | C | T | SNP | Intron | 548 | 98.6/1.4 | 60 |
| 9. | *SSI* | 3072 | G | A | SNP | Intron | 559 | 99.0/1.0 | 60 |
| 10. | *SSI* | 3138 | C | T | SNP | Intron | 556 | 100/0 | 60 |
| 11. | *SSI* | 3160 | T | A | SNP | Intron | 469 | 97.2/2.8 | 60 |
| 12. | *SSI* | 3224 | A | G | SNP | Intron | 461 | 98.9/1.1 | 60 |
| 13. | *SSI* | 3786 | T | \|- | Indel | Intron | 693 | 98.8/1.2 | 60 |
| 14. | *SSIIa* | 851 | C | A | SNP | Intron | 845 | 91.0/9.0 | 60 |
| 15. | *SSIIa* | 3797 | G | A | SNP | Exon | 762 | 94.0/6.0 | 60 |
| 16. | *SSIIa* | 3901 | T | G | SNP | Exon | 762 | 88.0/12.0 | 60 |
| 17. | *SSIIIa* | 1615 | C | T | SNP | Exon | 943 | 89.0/11.0 | 60 |
| 18. | *SSIIIa* | 2276 | T | C | SNP | Exon | 469 | 92.5/7.5 | 60 |
| 19. | *SSIIIa* | 3135 | C | A | SNP | Exon | 512 | 90.5/9.5 | 60 |
| 20. | *SSIIIa* | 5515 | G | A | SNP | Exon | 683 | 99.0/1.0 | 60 |
| 21. | *SSIIIa* | 9076 | C | A | SNP | Intron | 596 | 89.8/10.2 | 60 |
| 22. | *SSIIIa* | 9467 | T | C | SNP | Intron | 923 | 95.6/4.4 | 60 |
| 23. | *SSIIIa* | 9517 | T | C | SNP | Intron | 569 | 89.8/10.2 | 60 |
| 24. | *SSIIIa* | 10336 | G | A | SNP | Intron | 765 | 94.0/6.0 | 60 |
| 25. | *SBEIa* | 1137 | C | T | SNP | Intron | 567 | 98.8/1.2 | 60 |
| 26. | *SBEIa* | 2337 | C | T | SNP | Intron | 937 | 86.6/13.4 | 60 |
| 27. | *SBEIIb* | 1950 | C | - | Indel | Intron | 582 | 100/0 | 60 |
| 28. | *SBEIIb* | 2648 | T | G | SNP | Intron | 592 | 99.0/1.0 | 60 |
| 29. | *SBEIIb* | 2857 | T | G | SNP | Intron | 636 | 100/0 | 60 |
| 30. | *SBEIIb* | 9536 | A | G | SNP | Intron | 692 | 95.3/4.7 | 60 |
| 31. | *SBEIIb* | 10363 | A | C | SNP | Intron | 631 | 91.0/9.0 | 60 |
